# Scanless two-photon voltage imaging

**DOI:** 10.21203/rs.3.rs-2412371/v1

**Published:** 2023-01-24

**Authors:** Ruth R. Sims, Imane Bendifallah, Christiane Grimm, Aysha Mohamed-Lafirdeen, Xiaoyu Lu, François St-Pierre, Eirini Papagiakoumou, Valentina Emiliani

**Affiliations:** 1 Institut de la Vision, Sorbonne Université, INSERM, CNRS, F-75012 Paris, France; 2 Systems, Synthetic, and Physical Biology Program, Rice University, Houston, TX, USA; 3 Department of Neuroscience and Department of Biochemistry and Molecular Biology, Houston, TX, USA; 4 Department of Electrical and Computer Engineering, Rice University, Houston, TX, USA

**Keywords:** Two-photon microscopy, voltage imaging, temporal focusing, optogenetics, computer-generated holography

## Abstract

Parallel light-sculpting methods have been used to perform scanless two-photon photostimulation of multiple neurons simultaneously during all-optical neurophysiology experiments. We demonstrate that scanless two-photon excitation also enables high-resolution, high-contrast, voltage imaging by efficiently exciting fluorescence in a large fraction of the cellular soma. We present a thorough characterisation of scanless two-photon voltage imaging using existing parallel approaches and lasers with different repetition rates. We demonstrate voltage recordings of high frequency spike trains and sub-threshold depolarizations in intact brain tissue from neurons expressing the soma-targeted genetically encoded voltage indicator JEDI-2P-kv. Using a low repetition-rate laser, we perform recordings from up to ten neurons simultaneously. Finally, by co-expressing JEDI-2P-kv and the channelrhodopsin ChroME-ST in neurons of hippocampal organotypic slices, we perform single-beam, simultaneous, two-photon voltage imaging and photostimulation. This enables in-situ validation of the precise number and timing of light evoked action potentials and will pave the way for rapid and scalable identification of functional brain connections in intact neural circuits.

## Introduction

Deciphering the logic and syntax of neural computation is a central goal in neuroscience and requires methods to record (read-out) and manipulate (write-in) the activity of individual neurons. Electrophysiological methods have proven instrumental towards achieving this goal since they can read and write neural activity with high fidelity. However, while extracellular probes can record from large populations, they have limited spatial resolution and cannot excite or inhibit specific neurons. In contrast, whole-cell patch-clamp methods can manipulate and record the electrical activity of targeted neurons but are hard to achieve *in vivo* even for a handful of neurons simultaneously and are unsuitable for longitudinal (chronic) studies. Furthermore, all electrophysiological methods have limited access to smaller cellular compartments of neurons (such as axons, distal dendrites, spines and boutons). These limitations have stimulated the development of a plethora of minimally invasive photonic approaches, combining advanced optical methods with light-sensitive proteins, such as genetically encoded fluorescent indicators and optogenetic actuators, for recording and manipulating neural activity, respectively^1–3^.

In the nervous system, calcium ions regulate a broad range of processes and generate versatile intracellular signals^4^. Since action potentials lead to a large elevation of intracellular calcium, which can last an order of magnitude longer than the action potentials themselves^5^, the developments of synthetic^6^ and genetically encoded^7^ fluorescent calcium indicators (GECIs) capable of reporting changes in intracellular calcium were extremely important scientific breakthroughs. GECIs can be targeted to sub-cellular compartments and specific cell types^8,9^. Their long-term expression in intact tissues and organisms^2^ enables the repeated observation of individual cells. Calcium transients last significantly longer than the underlying voltage fluctuations, facilitating the detection of neural activity, but also limiting the quantification of spike firing rate and timing. Furthermore, GECIs are not well-suited for detecting sub-threshold voltage changes and hyperpolarizations resulting from synaptic and neuromodulatory inputs^10^.

Voltage indicators, which generate optical signals whose magnitude varies as a function of membrane potential, promise to address many of the aforementioned limitations of GECIs^11^. Following the first optical recordings of membrane potential with a synthetic dye^12^, voltage-sensitive indicators have undergone continual advancements, including improved synthetic dyes^13^, genetically encoded voltage indicators (GEVIs) and hybrid GEVIs^14^. However, detecting voltage spikes with GEVIs requires millisecond-timescale imaging, two orders of magnitude faster than generally required for GECIs. This technical challenge has limited the broad adoption of GEVIs for population imaging with cellular resolution.

The majority of voltage imaging experiments have relied on widefield, one-photon (1P) illumination and detection. The resulting mesoscopic observations of population activity have enabled investigation of the functional organisation and dynamics of cell-type specific excitatory and inhibitory cortical circuits^15,16^. The lack of optical sectioning of 1P widefield microscopy has been overcome using sparse labelling strategies^17^ or sculpted illumination^18–21^. However, these strategies are not suitable for multitarget voltage imaging in densely labelled scattering samples with cellular resolution, such as mammalian in-vivo preparations.

In principle, the optical sectioning inherent to two-photon excitation can be used to overcome these problems^22^, and two-photon laser scanning microscopy (2P-LSM) is commonly used to perform calcium imaging in scattering tissue^23^. However, the acquisition rate of conventional 2P-LSM is limited and millisecond transients such as action potentials can only be detected by drastic reduction of the field of view^24–27^. As a result, several specialised scanning-based techniques have been developed to image neural activity across larger areas at kilohertz rates^28–35^ and have yielded spectacular results, such as recording the voltage dynamics of cortical neurons in layer 5 in awake behaving mice. However, these methods are extremely technically demanding, have thus-far been limited to imaging a few cells simultaneously, and have not yet been demonstrated to be compatible with two-photon optogenetics, as required for two-photon, all-optical neurophysiology experiments.

Here, we propose an alternative approach for high-contrast, high-resolution, voltage imaging in densely labelled samples that is compatible with simultaneous two-photon optogenetic stimulation. Our method leverages existing scanless two-photon excitation approaches^36–39^ and the recently developed soma-targeted GEVI JEDI-2P-kv^40^. We demonstrate that, in combination with temporal focusing (TF)^41–43^, the three light-sculpting approaches commonly used for scanless two-photon photoactivation —Generalised Phase Contrast (GPC)^36,38^, low numerical aperture (NA) Gaussian beams (such as 3D-SHOT)^39,44^ and Computer-Generated Holography (CGH)^45,46^— enable voltage imaging in mammalian cells. By performing simultaneous imaging and electrophysiology, we provide a thorough quantitative comparison of these illumination modalities. Next, by viral expression of JEDI-2P-kv in mouse hippocampal organotypic slices, we show that 2P-TF-GPC enables high spatiotemporal resolution voltage imaging of neural activity in extremely densely labelled preparations. We further demonstrate the detection of high-frequency spike trains and subthreshold membrane depolarizations with amplitudes on the order of excitatory postsynaptic potentials (PSPs). Capitalising on the overlapping spectra of JEDI-2P-kv and the channelrhodopsin ChroME-ST^44^, we demonstrate simultaneous two-photon voltage imaging and photostimulation in multiple cells. This approach enables *in-situ* characterisation of the light-induced spiking properties of a population of neurons. Collectively, these results pave the way for studying neural function with two-photon all-optical neurophysiology in highly-scattering, densely labelled preparations.

## Results

### Scanless two-photon voltage imaging with sculpted, temporally focused excitation

**Figure 1:**
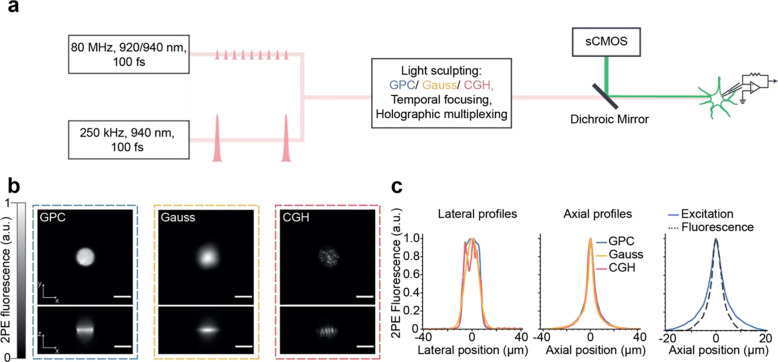
Schematic and characterization of the optical setup developed for scanless two-photon voltage imaging **(a)** Summary of the optical setup designed to generate 12 μm (Full Width Half Maximum), temporally focused, Gaussian, Generalised Phase Contrast (GPC) and holographic (CGH) spots. The setup was equipped with three lasers, two of them delivering nJ-pulse energies at 80 MHz (Coherent Discovery, 1 W, 80 MHz, 100 fs tuned to 920, 940 or 1030 nm; Spark Alcor, 4 W, 80 MHz, 100 fs, 920 nm) and the third a custom Optical Parametric Amplifier (OPA) pumped by an amplified fibre laser, with fixed wavelength output (Amplitude Satsuma Niji, 0.5–0.6 W, 250 kHz, 100 fs, 940 nm). Fluorescence signals were acquired using an sCMOS camera. The microscope was equipped for electrophysiology patch-clamp recordings. **(b)** Lateral and axial cross sections of two-photon excited fluorescence generated with Gaussian (yellow), GPC (blue) and GCH (red) beams, as indicated in the legend. Scale bars represent 10 μm. **(c)** Lateral and axial profiles of two-photon excited fluorescence generated with each excitation modality, and the corresponding system response, demonstrating single-cell resolution.

The optical setup (Figure 1a, Supplementary Figure 1 and Table S1) was comprised of two independent excitation paths, one designed to generate temporally focused (TF) Generalized Phase Contrast (GPC) patterns^36,38^ or low NA Gaussian spots (similar to 3D-SHOT^39^), and the second for TF-Computer Generated Holography (CGH)^37^. These paths were combined prior to the microscope objective with a polarising beam splitter. Each excitation path was designed to generate temporally focused spots with dimensions matching the typical size of a neuronal soma (12 μm lateral full width at half maximum (FWHM) and ~9 μm axial FWHM for all modalities (Figure 1b, c, Supplementary Note 2, Supplementary Figures 2–5)). The fluorescence from 1 μm microspheres excited with a 12 μm GPC spot was recorded and found to have an axial FWHM of 3.7 μm demonstrating sub-cellular axial resolution (Figure 1c, right panel). In all cases, emitted fluorescence was detected by an sCMOS camera, effective pixel size 0.1625 μm. The nominal field of excitation of each of the light sculpting approaches was 250 × 250 μm^2 6,34,35^. However, the effective imaging field of view was limited in one dimension by the number of sCMOS rows readout simultaneously at a given acquisition rate (see Methods). The system was equipped with three different laser sources, two high repetition rate oscillators (as commonly used for two-photon laser scanning microscopy; 80 MHz, 920 or 940 nm, 100 fs, 12.5 nJ and 50 nJ pulse energies) and a third low repetition rate, high pulse energy laser (250 kHz, 940 nm, 100 fs, up to 2.5 μJ energy per pulse).

**Figure 2:**
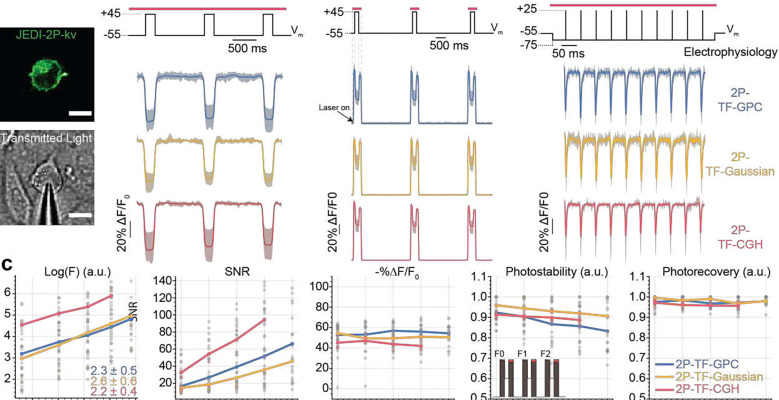
In-vitro electrophysiological characterisation of scanless two-photon voltage imaging in cultured CHO cells. **(a)** Confocal image of a JEDI-2P-kv expressing CHO cell (upper) and transmitted light image of a patched CHO cell (lower). Scale bars represent 10 μm. **(b)** Data from three protocols used to test the performance of each of the three different parallel illumination modalities for two-photon voltage imaging. Responses are reported as the fluorescence change (ΔF) normalized by the baseline fluorescence (F_0_), expressed as a percentage of the baseline fluorescence (%ΔF/F_0_). The average trace and 95 percent confidence interval from all cells imaged with each modality are plotted (blue – GPC, yellow – Gaussian, red – CGH). The corresponding electrophysiology control signals are plotted in black. The red bar above the electrophysiology trace indicates the illumination epoch. **(c)** Quantification of data for all cells from protocol 2. Log(F), SNR, −%ΔF/F_0_, photobleaching and photorecovery are plotted as a function of power density (power density: 0.66 – 1.55 mW μm^−2^, 75 – 175 mW per cell, n = 8 – 13), see also Supplementary Figure 9. Each point represents a measurement from an individual cell. The mean is plotted for each condition. Photostability is defined as the ratio between the integral of the baseline fluorescent trace to F_0_*n_t_ where F_0_ represents the fluorescence in the first frame and n_t_ the number of baseline fluorescence timepoints (see schematic diagram, fourth panel, inset). Photorecovery is defined as the average ratio of the fluorescence prior to the 100-mV depolarization in each illumination epoch (for instance F_1_/F_0_ as defined in the schematic diagram, fourth panel, inset). All data was acquired with laser A tuned to 940 nm and camera A (See Supplementary Figure 1 and Supplementary Tables 1 and 2).

We compared the performance of three excitation modalities (2P-TF-GPC, 2P-TF-Gaussian and 2P-TF-CGH) for scanless two-photon voltage imaging using a high repetition rate laser source, as typically used for conventional 2P-LSM. We transiently expressed a recently developed, negative-going, voltage indicator optimised for two-photon excitation (JEDI-2P-kv^40^), in mammalian (CHO) cells (Figure 2a). We controlled the membrane potential of individual cells using whole-cell patch-clamp electrophysiology and simultaneously performed two-photon voltage imaging. We used three different protocols, hereafter named 1, 2 and 3 (Figure 2b), to test the feasibility of scanless two-photon voltage imaging and to assess the advantages and disadvantages of each parallel approach.

Protocol 1 was used to quantify the voltage sensitivity of fluorescence of cells expressing JEDI-2P-kv. The responses of patched cells to three 100 ms, 100 mV voltage steps were recorded at 100 Hz under continuous illumination (power density: 0.88 mW μm^−2^, 100 mW per cell) for 3 seconds. Voltage responses were clearly observed as a decrease in fluorescence with 2P-TF-GPC, 2P-TF-Gaussian and 2P-TF-CGH (Figure 2b, left panel). For most cells, we observed variability between the response amplitude measured at different membrane locations. Since differences in voltage responsivity of the fluorescence originating from different portions of the membrane were random, this is plausibly due to differences in plasma membrane trafficking and protein folding.

All data acquired using protocol 1 (n = 41 cells) were pooled and used to establish and validate an analysis pipeline capable of automatically identifying and segmenting neurons and of detrending the optical traces (Supplementary Figure 6). Due to the similarities between the data obtained with scanless two-photon voltage imaging and single photon voltage imaging with widefield detection, it was possible to develop an analysis pipeline based on existing open-source packages. Compared with results obtained by calculating the unweighted mean of all pixels within segmented cells, the regression-based pixel weighting algorithm^14,49–51^ (Methods, Supplementary Note 3), which improved segmentation, was found to increase −%ΔF/F_0_ for all modalities (33.8 ± 9.5 vs. 43 ± 11.7, mean ± s.d., p < 0.00001, n=41, Supplementary Figure 7b), resulting in values in accordance with those previously reported^40^. No significant difference in SNR (signal amplitude divided by the standard deviation of the baseline signal) was found between the two approaches (59.4 ± 30.2 vs. 59.6 ± 31.3, p = 0.9158, n=41, Supplementary Figure 7c), a result of the fact that the improved segmentation contained approximately half of the pixels of the initial segmentation (16263 ± 2180 vs. 8962 ± 2676, p < 0.00001, n=41, Supplementary Figure 7d). However, the location of these pixels coincided with the exterior cell membrane (Supplementary Figure 7d, inset), the most voltage-sensitive, which compensated for the effective reduction in photon count. We found that the final traces generated using the weighted pixel mask exhibited slightly more photobleaching than the traces generated with the original segmentation (0.82 ± 0.02 vs. 0.80 ± 0.03, p < 0.00001, n=41, Supplementary Figure 7e). This is likely the result of two factors. Firstly, responsive pixels imaged with high contrast are more likely to be retained in the second segmentation step. These pixels are those where the cellular equator coincided with the focal plane, where the excitation power density (and presumably photobleaching) is highest. Secondly, the voltage responsive fluorophores are more likely to be tethered to the membrane, less mobile and hence more susceptible to photobleaching.

Having established the analysis pipeline, we then compared the three excitation modalities (Supplementary Figure 8). 2P-TF-GPC, 2P-TF-Gaussian and 2P-TF-CGH were all found to be suitable for scanless two-photon voltage imaging. Data obtained with 2P-TF-CGH exhibited the highest signal-to-noise ratio (81.6 ± 35.3, n = 15), almost double that of 2P-TF-GPC (48.5 ± 19.2, p = 0.00222, n = 17) and 2P-TF-Gaussian (43.9 ± 20.3, p = 0.00608, n = 9). We hypothesised this was because the high spatial density of photons in speckle grains results in more efficient two-photon excitation. This hypothesis was confirmed by simulations (Supplementary Note 2, Supplementary Figure 5).

Since the high density of photons in speckles also increase the likelihood of non-linear photophysics (for instance photobleaching^52^), we designed and used a different protocol (Protocol 2) to investigate the extent of these non-linear effects as a function of the excitation power density (0.66 – 1.55 mW μm^−2^ corresponding to 75 – 175 mW per cell). Protocol 2 consisted of three 100 ms, 100 mV voltage steps, 200 ms illumination pulses centred on each voltage step, and 2.5 s inter-pulse intervals (Figure 2b, middle panel). For all modalities, the baseline fluorescence (F_0_) increased quadratically as a function of power density (Figure 2c, first panel), as expected with two-photon illumination and indicating that fluorescence excitation was not saturated at any of the powers used. Furthermore, the SNR increased linearly as a function of power density (Figure 2c, second panel, *R*^2^ = 0.999 (2P-TF-GPC), *R*^2^ = 0.995 (2P-TF-Gaussian), *R*^2^ = 0.998 (2P-TF-CGH)), confirming that experiments were performed in the shot-noise limited regime rather than being limited by the read noise of the detector. On this basis, all SNR estimates stated hereafter were calculated as SNR = (−ΔF/F_0_)√ F_0_^53^.

The SNR of the responses to 100 mV steps (Protocol 2) was higher at all excitation power densities with 2P-TF-CGH than with 2P-TF-GPC or 2P-TF-Gaussian, (Figure 2c, second panel, Supplementary Figure 9a), though the 2P-TF-CGH fluorescent transients exhibited a systematically lower average −%ΔF/F_0_ than when using 2P-TF-GPC. This difference increased as a function of power density (Figure 2c, third panel, Supplementary Figure 9b). Photostability, defined as the ratio between the integral of the baseline fluorescent trace to F_0_*n_t_ where F_0_ represents the fluorescence in the first frame and n_t_ the number of baseline fluorescence timepoints (schematic diagram, Figure 2c, fourth panel, inset), decreased as a function of excitation power in all cases (Figure 2c, fourth panel). No significant difference was observed between the different modalities (Supplementary Figure 9c). Photorecovery, quantified as the ratio of fluorescence after dark intervals to the original fluorescence (schematic diagram, Figure 2c, fourth panel, inset), was over 97% following 2.5 s dark inter-pulse intervals when excited with 2P-TF-Gaussian and 2P-TF-GPC, which is consistent with previous observations^28^. In the case of 2P-TF-CGH, the photorecovery decreased as a function of excitation power density and was lower than the other modalities (<95% for power densities greater than 0.88 mW μm^−2^ (100 mW per cell), p=0.01, Figure 2c, fifth panel, Supplementary Figure 9d).

Finally, we used protocol 3 (Figure 2b, right panel) to assess the detection of short, action-potential like transients with scanless two-photon voltage imaging. Cells were illuminated continuously for 500 ms (power density: 1.33 mW μm^−2^, corresponding to 150 mW per cell) and the fluorescence response to a 20 Hz train of 10 rectangular pulses (100 mV amplitude, 3 ms duration) was recorded with a 1 kHz acquisition frequency (see Methods). The transients recorded using 2P-TF-GPC, 2P-TF-Gaussian and 2P-TF-CGH, had −%ΔF/F_0_ values of 45 ± 14 %, 42 ± 16 % and 26 ± 6 % (n = 8–11) respectively (Supplementary Figure 10b). For all modalities, the average SNR was greater than 11, demonstrating that action-potential-like signals can be reliably detected in single trials with scanless two-photon voltage imaging. As per data presented in supplementary figures 10c and d, the highest SNR data was acquired using 2P-TF-CGH (20.5 ± 6.2 compared with 13.9 ± 3.6 (2P-TF-GPC) and 11.5 ± 4.1 (2P-TF-Gaussian), n = 8–11), at the cost of lower photostability (0.86 ± 0.07 (2P-TF-CGH) versus 0.92 ± 0.08 (2P-TF-GPC) and 0.89 ± 0.12 (2P-TFGaussian), n = 8–11).

Overall, these results confirm that 2P-TF-GPC, 2P-TF-Gaussian and 2P-TF-CGH can successfully be applied to scanless two-photon voltage imaging, albeit with different advantages and limitations. Since the SNR of data acquired using 2P-TF-CGH was significantly higher than for 2P-TF-Gaussian or 2P-TF-GPC, we consider it the optimal modality for imaging large numbers of cells simultaneously, for short periods, with a given incident power. For prolonged recordings (continuous illumination for hundreds of milliseconds or more) of neurons labelled with JEDI-2P-kv, we would recommend 2P-TF-GPC or 2P-TF-Gaussian, since we observed lower photobleaching and higher photorecovery with these methods than with 2P-TF-CGH. Although no significant performance differences were found between 2P-TF-Gaussian and 2P-TF-GPC (Figure 2c, Supplementary Figures 8 and 9), 2P-TF-Gaussian requires higher power at the laser output for a given SNR. Specifically, uniform illumination of the somal membrane was achieved with 2P-TF-Gaussian by expanding and subsequently cropping the beam (Supplementary Note 2, Supplementary Figure 4) and thus was ~3 times less power-efficient than 2P-TF-GPC^54^. However, it is perhaps the simplest approach to implement, and hence a good solution given a sufficiently powerful laser source.

### Scanless voltage imaging of neural activity in hippocampal organotypic slices with two-photon, temporally focused Generalised Phase Contrast

**Figure 3:**
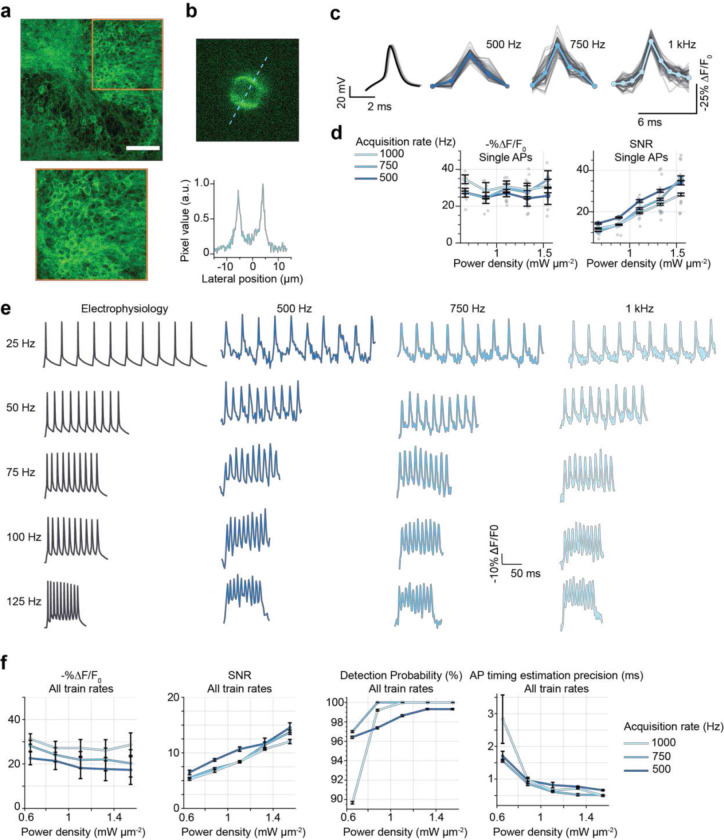
Recording electrically evoked single action potentials and high-frequency spike trains in JEDI-2P-kv expressing hippocampal organotypic slices with 2P-TF-GPC. **(a)** Upper: confocal image of a representative organotypic slice bulk-infected with JEDI-2P-kv. Scale bar represents 75 μm. Lower: zoom (x2) of densely expressing region where data was recorded. **(b)** Upper: representative single frame from data acquired with TF-GPC (1 ms exposure time), Lower: line-profile through the image (indicated by the dashed line) demonstrating that single cells are imaged with high-contrast in densely labelled samples with 2P-TF-GPC. **(c)** Electrically induced and recorded action potentials (left) and optically recorded (right) were resolved in single trials using 2P-TF-GPC at different acquisition rates. Individual trials are plotted in grey. The average trace across all trials is plotted in a different shade of blue corresponding to each acquisition rate (500 Hz, 750 Hz and 1 kHz, as labelled). Power density: 1.1 mW μm^−2^ (125 mW per cell). **(d) −**%ΔF/F_0_ and SNR plotted as a function of power density in different shades of blue for different acquisition rates (see legend). Error bars represent the standard error of measurements across all cells (n = 4–6). Individual points represent the average value over 50 action potentials for individual cells. All data were acquired using laser A tuned at 940 nm, and camera A (See Supplementary Figure 1 and Supplementary Tables 1 and 2). **(e)** Representative fluorescence traces recorded from individual cells to different rates of electrically evoked spike trains recorded at the different acquisition rates of 500 Hz, 750 Hz and 1 kHz corresponding to 2 ms, 1,33 ms and 1 ms exposure time (power density: 1.1 mW μm^−2^, 125 mW per cell). A representative trace of electrically evoked spike trains is also plotted in black (left). **(f)** −%ΔF/F_0_, SNR, action potential detection probability and precision of action potential timing estimation (defined as the jitter in timing estimation for all identified action potentials relative to the corresponding electrophysiological recordings) plotted as a function of power density for different acquisition rates (500 Hz, 750 Hz, and 1 kHz, see legend). A lower value indicates superior timing estimation. Data plotted for all train rates (n = 2–5). All data were acquired using laser B fixed at 920 nm, and camera B (See Supplementary Figure 1 and Supplementary Tables 1 and 2

We set out to identify the imaging conditions (specifically the power densities and acquisition rates) required to observe neural activity ranging from high-frequency spike trains to sub-threshold depolarizations in densely labelled samples. We also aimed to determine whether the necessary imaging conditions perturb neural activity or otherwise impact cellular physiology. We performed simultaneous 2P-TF-GPC imaging and whole cell-patch clamp recordings of granule cells located in the dentate gyrus (DG) of organotypic slices bulk-transduced with JEDI-2P-kv (see Methods). Expression of JEDI-2P-kv in the granule cells of the DG was well localised to the plasma membrane, with no evidence of intracellular aggregation (Figure 3a). Even though granule cells are extremely closely packed in DG, due to the optical sectioning conferred by temporally focused, targeted illumination, we were able to image individual neurons with high-contrast and high-resolution in this challenging preparation (Figure 3b).

Using protocol 1, we confirmed we could detect 100 mV depolarizations in densely labelled, scattering organotypic slices with comparable −%ΔF/F_0_ (43 ± 8) to that obtained in CHO cells (51 ± 11) (n > 15 cells, accounting for differences in the resting potential between neurons and CHO cells, Supplementary Figures 11 and 12). No significant difference was observed in SNR (69 ± 25 (CHO), 50 ± 30 (organotypic slices), n > 15 cells, Supplementary Figure 12e) or photostability between results obtained in hippocampal organotypic slices and CHO cells. The effective lateral and axial resolution of the scanless two-photon imaging system, quantified as the relative ΔF/F_0_ of an electrically evoked spike as a function of the distance between the excitation spot and the soma, was found to be approximately isotropic and of similar dimensions to the neuronal soma (14 μm lateral and 13 μm axial FWHM, Supplementary Figure 12h), confirming the cellular resolution of scanless two-photon voltage imaging.

Next, we recorded the fluorescence from patched cells while 50 action potentials (APs) were evoked electrically by injection of current (700 – 900 pA, 2 ms) into the soma at a rate of 1 Hz. Electrically evoked APs were imaged with 3 different acquisition rates: 500 Hz, 750 Hz, and 1 kHz (corresponding to per-frame exposure times of 2, 1.33 and 1 ms respectively) as previously used for 1P widefield voltage imaging^21^. In all conditions, individual APs could clearly be identified from single trials in the raw fluorescence traces (representative traces for single cells plotted in Figure 3c, power density: 1.11 mW μm^−2^, corresponding to 125 mW per cell). Putative APs were identified by template matching, based on the most prominent peaks originally identified in each fluorescence trace^50^. The 1 kHz recordings exhibited a higher −%ΔF/F_0_ than 500 Hz recordings across all powers (30.5 ± 2.2 vs 25.7 ± 1.2, n > 5 cells, Figure 3d). However, consistent with previous reports^17^, higher SNR was achieved with 500 Hz recordings than for 1 kHz (for example for 0.66 mW μm^−2^ (75 mW per cell): 14.2 ± 0.3 vs 11.9 ± 0.3, Figure 3d), because the increase in the number of photons collected per action potential more than compensated for the reduced −%ΔF/F_0_.

Having established that it was possible to record APs with high SNR in single trials at different acquisition rates, we next tested whether we could also monitor individual spikes within high-frequency trains of action potentials (such as bursts) under these conditions. We observed that an acquisition rate of 500 Hz was sufficient to track individual APs in trains with frequencies up to 100 Hz (Figure 3e, Supplementary Figure 13) and using power densities as low as 0.66 mW μm^−2^ (75 mW per cell). As a result of increased SNR recordings with lower acquisition rates, at low power densities, the detection probability (fraction of correctly identified APs) was higher (Figure 3f). Note that the difference in SNR between results presented in Figure 3d and Figure 3f is the result of using different cameras and different excitation wavelengths, as specified in the Methods and Supplementary Tables 1 and 2. However, an acquisition rate of 500 Hz was insufficient for robustly tracking spikes in 125 Hz trains due to a reduction in −%ΔF/F_0_ (Supplementary Figure 13b), which led to a deterioration in detection probability and fluorescence response compared with the data acquired at 1 kHz at power densities ≥ 0.66 mW μm^−2^ (corresponding to 75 mW per cell, Figure 3f, Supplementary Figure 13). For all power densities > 0.66 mW μm^−2^ (75 mW per cell) sub-millisecond precision of AP timing estimation was obtained, as measured with respect to the electrophysiology trace (Figure 3f).

**Figure 4:**
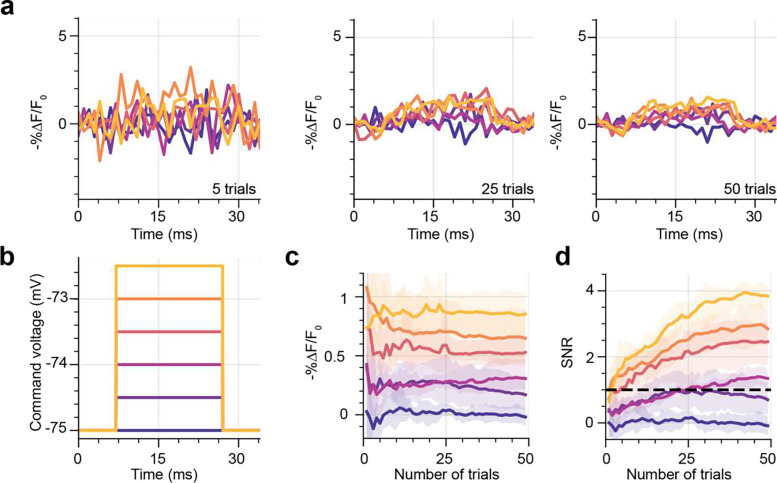
Recording sub-threshold depolarizations in JEDI-2P-kv expressing hippocampal organotypic slices using 2P-TF-GPC. **(a)** Average fluorescence traces recorded from neurons after 5, 25 and 50 trials for different magnitudes of sub-threshold depolarizations ranging between 0 and 2.5 mV. Sub-threshold depolarisations < 2.5 mV cannot be reliably resolved in single trials using 2P-TF-GPC and JEDI-2P-kv, however after 25 trials depolarisations greater than or equal to 1 mV can be resolved. Traces were recorded with a 1 ms exposure time and 1.1 mW μm^−2^ (125 mW per cell). **(b)** Command voltage steps used to change the membrane potential of patched neurons. **(c)** Average −%ΔF/F_0_ and **(d)** SNR of the fluorescence response to different sub-threshold changes of membrane potential plotted as a function of number of repeats. The 95% confidence interval is also plotted (shaded region). All data (n = 6) were acquired using laser B fixed at 920 nm and camera B (See Supplementary Figure 1 and Supplementary Tables 1 and 2).

We next examined whether these conditions (power density: 1.11 mW μm^−2^, corresponding to 125 mW per cell, 1 kHz acquisition rate) were also suitable for imaging sub-threshold changes in membrane potential. To emulate excitatory PSPs, patched cells were clamped to −75 mV, while the membrane potential was varied in 0.5 mV steps from 0 to 2.5 mV for 20 ms. This protocol was repeated 50 times. Since it was not possible to detect these transients from individual recordings (Figure 4a, b, n=6), we averaged data from different trials to improve SNR. Averaging data from 25 repeats was sufficient to stabilise the magnitude of the fluorescence transient (−%ΔF/F_0_) for a given depolarization (Figure 4a, c) and to increase the SNR above 1 for all depolarizations larger than 0.5 mV (Figure 4d).

**Figure 5:**
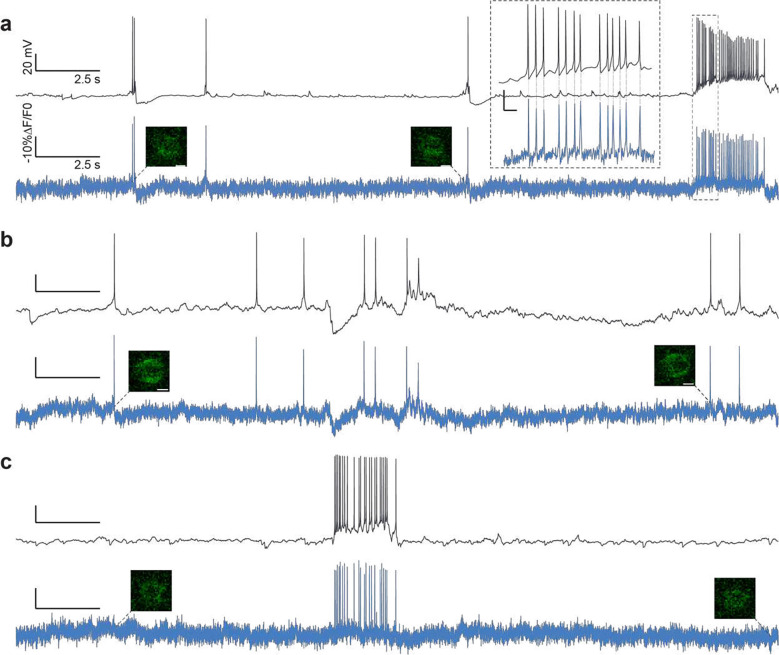
Recording spontaneous neural activity in JEDI-2P-kv expressing hippocampal organotypic slices using 2P-TF-GPC **(a-c)** Simultaneous current clamp (upper, black) and fluorescence recordings (lower, blue) of spontaneous activity in neurons from hippocampal organotypic slices over a continuous 30 s recording period. Single imaging frames are shown close to the beginning and end of each recording. Scale bars represent 5 μm. Inset, **(a)** zoomed in portion of the electrophysiological and fluorescence traces. Corresponding action potentials in the electrophysiological and fluorescence traces (average rate: 17 Hz) is indicated by the dashed lines. (Power density: 1.33 mW μm^−2^, 150 mW per cell, 1 kHz acquisition rate). All data was acquired using laser A tuned to 940 nm and camera A (See Supplementary Figure 1 and Supplementary Tables 1 and 2).

Next, we tested the capability of scanless two-photon voltage imaging to record spontaneous network activity, a fundamental feature of developing neural circuits^54^. We performed simultaneous electrophysiological (whole cell patch clamp, (current clamp)) and fluorescence recordings (2P-TF-GPC, power density: 1.33 mW μm^−2^ (150 mW per cell), 1 kHz acquisition rate) of spontaneous activity from neurons in hippocampal organotypic slices which exhibited a range of different resting potentials (n > 10 cells; 5 slices). We were able to observe several hallmarks of spontaneously generated activity, large slow depolarizations, bursts of action potentials, rhythmic sub-threshold depolarizations and hyperpolarizations (Figure 5 and Supplementary Figure 14). These results confirmed the capability of scanless two-photon voltage imaging for high temporal precision, single trial recordings of action potentials and sub-threshold events, even though the sensitivity curve of JEDI-2P-kv is not optimized for sub-threshold recordings. Collectively, the results presented in Figures 3–5 parameterize the necessary imaging conditions required to detect neural activity with sufficient SNR using scanless two-photon voltage imaging.

To test the robustness of our approach for long-term recordings, we repeated the same protocol (2P-TF-GPC, 30 s continuous illumination, 1.33 mW μm^−2^ (150 mW per cell), 1 kHz acquisition rate), for a maximum recording time of 20 minutes. Due to limitations of the prototype experimental configuration (primarily data transfer rates), there was a dark period (<10 seconds) between consecutive acquisitions. To increase the duty cycle of the recordings, we reduced the acquisition speed to 500 Hz which enabled us to perform longer continuous recordings with a shorter dark period of <5 seconds (2P-TF-GPC, 1 min continuous illumination, 1.33 mW μm^−2^ (150 mW per cell), 500 Hz acquisition rate). As indicated by the data presented in Supplementary Figure 15, we were able to record spontaneous activity from single neurons for a maximum recording time of 20 minutes without a significant decrease in SNR (Supplementary Figure 15b). The imaging period was primarily limited by axial sample drift which decreased the SNR (data not shown), but could be overcome in future experiments with a tandem construct containing the voltage indicator and a spectrally shifted, fluorescent reporter used to track the sample drift and to dynamically update the co-ordinates of the multiplexed spots.

Having established the conditions necessary to observe neural activity ranging from high-frequency trains of action potentials to sub-threshold membrane potential depolarizations, we next investigated whether such imaging conditions induced physiological perturbations. There are two main sources of light-induced perturbations. The first is heating, due to linear absorption of the infrared light (mostly by water), which has been reported to affect ion channel conductances^45^ and action potential waveforms^46^. The second is non-linear photodamage, due to higher order light-matter interactions which occur because of high instantaneous photon density in the focal volume and can ultimately induce apoptosis and cell ablation^47– 49^. We performed experiments (10 ms strobed illumination, 50 cycles, 1 Hz, total illumination time 500 ms, >15 cells per region, targeted sequentially: identical to the protocol used to detect APs (Figure 3b–c)) at power densities we found necessary to observe neural activity with sufficient SNR and above (0.66 – 1.55 mW μm^−2^, corresponding to 75 – 175 mW per cell). Following these experiments, we used immunohistochemistry to detect heat-shock proteins (anti-HSP70/72 immunostaining) and activation of apoptotic pathways (anti-activated-Caspase-3 immunostaining). Fixed slices were imaged using confocal microscopy. No difference in fluorescence intensity was observed between any of the illumination powers used and the control slices (not illuminated) in the case of Caspase-3 (Supplementary Figure 16a). In contrast, we observed that levels of anti-HSP increased as a function of excitation power above 1.1 mW μm^−2^ (125 mW per cell), which indicates that the physiological damage induced by the high repetition rate laser sources, is predominantly heating. Since the damage threshold of 1.1 mW μm^−2^ (125 mW per cell) identified using immunohistochemistry is an upper bound, we also investigated whether there was any light induced changes in the electrical properties of neurons using electrophysiology. We did not observe any light-induced changes in action potential amplitude or width at any of the tested powers (Supplementary Figure 16b), however we found that the latency of action potential firing slightly increased at all powers tested (0.1 ms, Supplementary Figure 16b). This effect was observed 15 seconds before a similar increase in latency was seen in control experiments (Supplementary figure 16b). Whilst a 10 percent change is not huge, the immunohistochemistry and electrophysiology results imply a laser-induced perturbation of physiology for powers > 125 mW per cell.

### Scanless two-photon voltage imaging of multiple targets with low repetition rate lasers

To test the capability of multiplexed 2P-TF-GPC to image multiple neurons simultaneously we used a custom low-repetition rate source (940 nm, pulse duration 100 fs, repetition rate 250 kHz, 600 mW average output power). Low-repetition rate sources, used for two-photon optogenetics, can provide higher peak energies and lower average power, hence potentially minimize photoinduced thermal effects and scale up the number of neurons that can be imaged simultaneously. Low-repetition rate lasers are particularly well suited for techniques with long times, such as scanless two-photon imaging. We found that using the low repetition rate laser, action potentials could be detected in single trials with power densities as small as 0.01 mW μm^−2^ (1.5 mW per cell, Supplementary Figure 17). In contrast to the results obtained using the high repetition rate source (80 MHz, previous section), no changes in action potential properties were detected at any of the tested powers (Supplementary Figure 17). Immunohistochemistry targeted against HSP70/72 and activated-Caspase-3 (Supplementary Figure 17) did not reveal thermal or non-linear damage at power densities below 0.09 mW μm^−2^ (10 mW per cell) using 2P-TF-CGH, two-fold higher than the maximum powers we found typically necessary to image neural activity. Extrapolating from the results related to photobleaching and photostability obtained in CHO cells, we anticipate higher non-linear damage thresholds for 2P-TF-GPC and 2P-TF-Gaussian.

**Figure 6:**
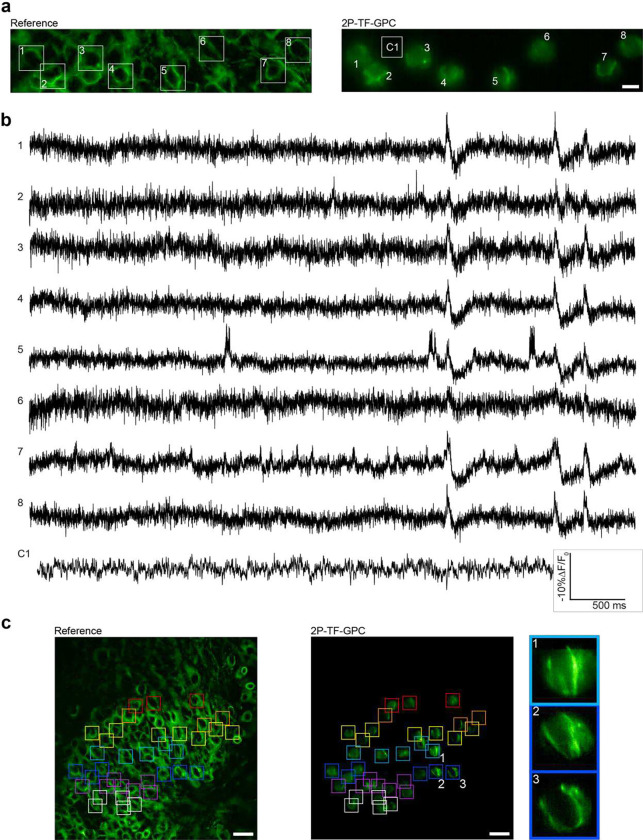
Multi-cell recordings of spontaneous neural activity in JEDI-2P-kv expressing hippocampal organotypic slices using multiplexed 2P-TF-GPC **(a)** Reference image of a hippocampal organotypic slice expressing JEDI-2P-kv in the dentate gyrus (left panel) and average projection of the corresponding voltage imaging dataset (right panel). 8 neurons targeted simultaneously with 8, 12-μm 2P-TF-GPC spots can be identified (as numbered and highlighted by the square boxes). The scale bar represents 10 μm. C1 refers to the area used to generate the control trace plotted in (b). This ROI was not targeted with a GPC spot during experiments. **(b)** Fluorescent traces plotted for each of the neurons indicated in (a), including the control trace. **(c)** Voltage imaging throughout a large field of view using multiplexed 2P-TF-GPC. Left panel: cross-section of a hippocampal organotypic slice expressing JEDI-2P-kv in the dentate gyrus. Middle panel: combined maximum projections of data from 7 consecutive acquisitions (indicated by the coloured squares), spanning a total area of 200 × 150 μm². Zoom in for best viewing. Scale bars represent 25 μm. Right panel: zoomed in regions of the central panel (indicated by numbering and coloured boxes) showing maximum projections of data acquired from individual cells targeted with multiplexed 2P-TF-GPC. All data was acquired using laser C (940 nm, power density: 0.02 – 0.09 mW μm^−2^, 2.5 – 10 mW per cell) and camera A with an acquisition rate of 1 kHz (See Supplementary Figure 1 and Supplementary Tables 1 and 2).

As a result of the increased energy per pulse of the low-repetition rate source, we were able to increase the number of neurons imaged simultaneously without exceeding the power damage threshold. For example, we recorded spontaneous activity in up to 8 neurons in the dentate gyrus of hippocampal organotypic slices simultaneously (2P-TF-GPC, power densities: 0.04 – 0.08 mW μm^−2^ (5 – 9 mW per cell, total power < 75 mW), Figure 6, Supplementary Figure 18). The sub-threshold activity of most neurons was found to be highly synchronized, a characteristic feature of the immature hippocampus^54^. Control traces recorded adjacent to targeted neurons confirmed that this was not an artefact due to crosstalk. We were able to combine data from separate acquisitions to perform voltage imaging throughout a large region (200 × 150 μm²) as demonstrated in Figure 6. Since this data was acquired on a prototype system, the period between sequential acquisitions was on the order of seconds. However, by optimizing the acquisition pipeline, the period between sequential acquisitions could feasibly be reduced to milliseconds to enable scanless two-photon voltage imaging of populations of neurons.

The number of achievable targets per acquisition and/or the imaging depth could be further increased, as demonstrated for two-photon photostimulation of multiple cells^55^ using high-power, low repetition rate, industrial light sources such as Ytterbium-doped fibre lasers which can provide much higher output powers (tens of Watts), and comparable pulse energies to the 940 nm source used in this work. These lasers are commonly fixed wavelength sources (1030 – 1040 nm), which means that they are not typically compatible with GFP-based fluorescent indicators. However, since the excitation spectrum of JEDI-2P-kv is slightly red-shifted as compared with previous GFP-based voltage indicators^40^, we tested whether it was possible to record neural activity using scanless two-photon voltage imaging with 1030 nm excitation. We repeated protocol 2 (see above) in CHO cells and imaged electrically evoked action potentials and spontaneous activity in sparsely labelled hippocampal organotypic slices (Supplementary Figure 19).

### Scanless two-photon voltage imaging and photostimulation of multiple targets with a single beam

**Figure 7:**
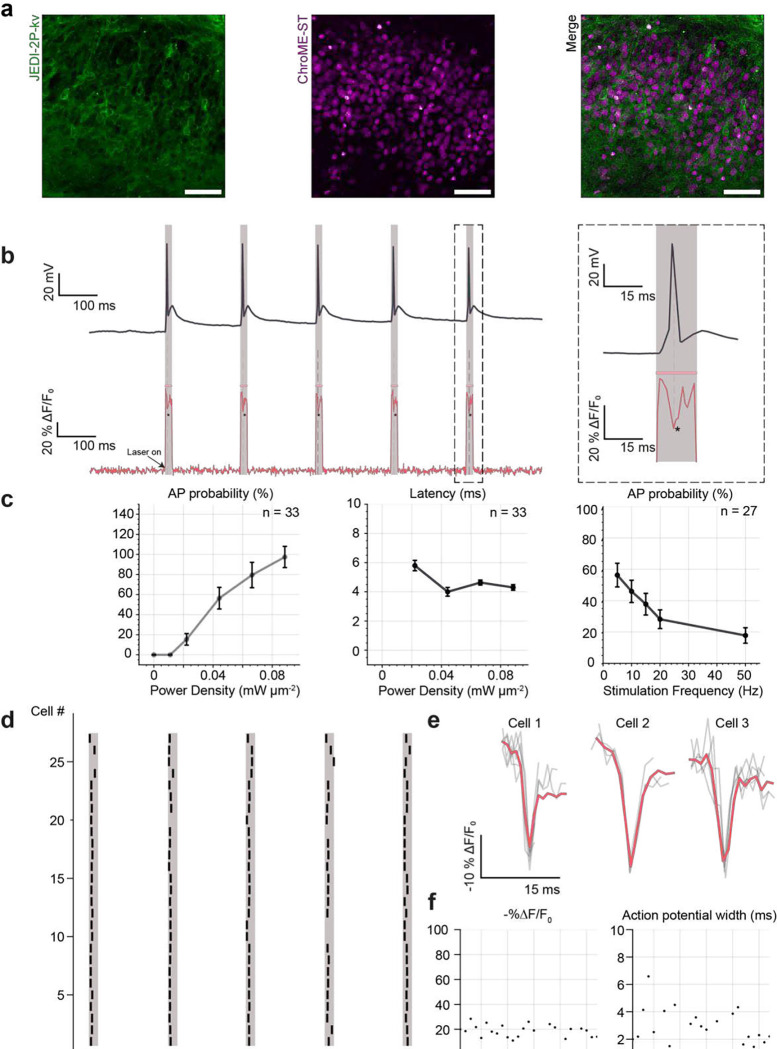
Fluorescence recordings of photo-evoked spikes in hippocampal organotypic slices co- expressing JEDI-2P-kv and ChroME-ST, using 2P-TF-CGH. **(a)** Cross-sections of hippocampal organotypic slices co-expressing the genetically encoded voltage indicator JEDI-2P-kv and the soma-targeted channelrhodopsin ChroME-ST in the dentate gyrus. Channelrhodopsin-expressing cells were identified according to their nuclear-localized fluorescence (see Methods). Scale bar represents 50 μm. **(b)** (left) Simultaneous optical and electrophysiological recordings demonstrating that action potentials can be evoked and imaged using a single excitation spot (12 μm diameter, power density 0.02 mW μm^−2^ (2.5 mW per cell), 15 ms strobed illumination at 5 Hz). (right) Zoom on simultaneous optical and electrophysiological recordings of one action potential. **(c)** All-optical in-situ characterisation of photo evoked action potentials. Error bars represent the standard error of recordings obtained for 33 cells. The probability of evoking and recording action potentials is plotted as a function of power density. Only cells in which at least one optically evoked action potential was detected are included. The latency of optically evoked action potentials is plotted as a function of power density. The average latency measured all-optically matches that obtained using electrophysiology (Supplementary Figure 20c). The action potential probability is plotted as a function of stimulation frequency. Action potential probability is calculated as the number of action potentials evoked and recorded over five trials (power density: 0.01 – 0.09 mW μm^−2^, 1.5 – 10 mW per cell). Error bars represent the standard error of recordings obtained for 33 cells. **(d)** Raster plot of 27 cells showing the number and timing of optically evoked action potentials (black) relative to the imaging/photostimulation epoch (red) (power density: 0.02 – 0.08 mW μm^−2^, 2.5 – 9 mW per cell). **(e)** Examples of fluorescence recordings of optically evoked action potentials for three representative cells. Individual trials are plotted in grey. The average trace across all trials is plotted in red (power density: 0.02 – 0.08 mW μm^−2^, 2.5 – 9 mW per cell). **(f)** Summary statistics for the amplitude (−%ΔF/F_0_) and width of the optically evoked action potentials from (d). All data were acquired using laser C fixed at 940 nm and camera A (See Supplementary Figure 1 and Supplementary Tables 1 and 2).

Next, we performed simultaneous two-photon voltage imaging and photostimulation of neurons co-expressing JEDI-2P-kv and a soma-targeted channelrhodopsin. ChroME-ST and JEDI-2P-kv were co-expressed in the dentate gyrus of hippocampal organotypic slices by bulk transduction of two Adeno-Associated Virus (AAV) vectors (Figure 7a). We characterised the photophysical properties of ChroME-ST excited using the low-repetition rate laser using whole cell patch clamp electrophysiology (voltage-clamp). ChroME-ST mediated photocurrents in CHO cells saturated at 0.02 mW μm^−2^ (2.5 mW per cell) (Supplementary Figure 20, n=4). In hippocampal organotypic slices, power densities between 0.02 and 0.04 mW μm^−2^ (2.5 – 5 mW per cell) generated sufficiently large photocurrents to reliably evoke APs with short (< 5 ms) latency and sub-millisecond jitter (Supplementary Figure 20, n=7).

We performed simultaneous photostimulation, imaging and whole-cell patch clamp recordings on neurons co-expressing ChroME-ST and JEDI-2P-kv and confirmed that optically evoked action potentials could be detected in voltage imaging recordings (Figure 7b). We next performed an all-optical characterisation of ChroME-ST. By modifying the power, duration and frequency of the illumination, we explored the joint-parameter space of imaging and stimulation conditions to optimize the probability of optically evoking and recording action potentials (Figure 7c). Unlike in neurons exclusively expressing JEDI-2P-kv, at power densities below 0.02 mW μm^−2^ (2.5 mW per cell), the SNR of action potentials from single trials did not exceed the SNR threshold and hence could not be detected optically (Figure 7c), although electrophysiological recordings performed simultaneously indicated that the probability of optically evoking an AP was greater than 75% (Supplementary Figure 20b). We attribute the reduction in SNR to a reduction in the expression efficiency of JEDI-2P-kv as a result of co-expressing a voltage indicator and channelrhodopsin, which results in a difference between the optical and electrophysiological results. However, for power densities above 0.02 mW μm^−2^ (2.5 mW per cell), we were able to detect action potentials in single trials and measured similar latencies to those obtained using whole-cell patch clamp recordings (4.3 ± 0.2 ms, mean ± s.e.m.) and jitter on the order of a millisecond (Supplementary Figure 20c-d).

The action potential probability decreased as a function of stimulation frequency, feasibly a result of channelrhodopsin desensitization at the saturating powers used, although in some cases it was possible to stimulate and image action potentials at 50 Hz (Figure 7c, right panel). Based on this characterisation, we determined that the optimal photostimulation and imaging parameters to robustly optically evoke and detect action potentials were a frequency of 5 Hz and 15 ms photoactivation pulses. The extended illumination time relative to typical photostimulation protocols was necessary for having sufficient baseline to calculate −%ΔF/F_0_ and to robustly detect optically evoked action potentials. Results obtained for these parameters are summarized for 27 cells in the raster plot in Figure 7d with representative fluorescence traces of optically evoked APs shown in Figure 7e. For each cell, the power density was increased until a spike was detected optically in at least one of five repeats. The final set of power densities used was between 0.02 and 0.08 mW μm^−2^ (2.5 and 9 mW per cell), below the power threshold found to induce physiological perturbations (see above). The average −%ΔF/F_0_ of optically evoked action potentials was found to be 20 ± 8 % (n=33; Figure 7f), consistent with results obtained for electrically evoked action potentials (Figures 3d and f).

**Figure 8:**
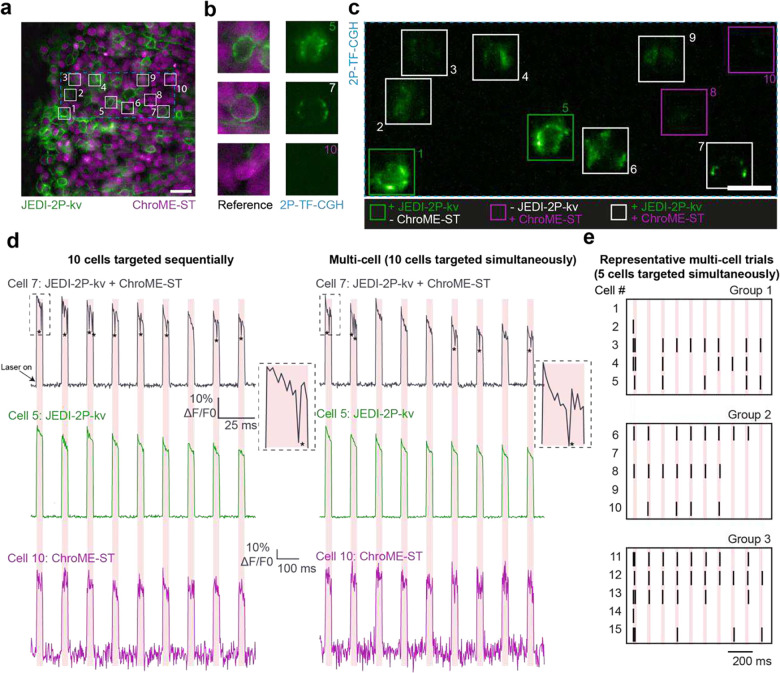
Characterisation of simultaneous multi-target photostimulation and voltage imaging using a single beam scanless two-photon excitation. **(a)** Cross-sections of hippocampal organotypic slices co-expressing JEDI-2P-kv and ChroME-ST in the dentate gyrus. The boxes indicate cells that were targeted simultaneously during a representative experiment (as numbered). Scale bar represents 25 μm. **(b)** Reference widefield images of individual targeted cells (left) and corresponding images obtained using 2P-TF-CGH. Upper: a cell exclusively expressing JEDI-2P-kv. Middle: a cell co-expressing JEDI-2P-kv and ChroME-ST. Lower: a cell exclusively expressing ChroME-ST. **(c)** Data acquired when the 10 cells identified in (a) were targeted simultaneously using 2P-TF-CGH and imaged at 500 Hz. Scale bar represents 15 μm. **(d)** Traces from the three cells highlighted in (b) when targeted sequentially (left) or simultaneously (right). Of the three selected cells, as expected, no action potentials were detected for cell 5 (green) or cell 10 (purple), which did not co-express the two constructs. In both the sequential and multi-cell acquisitions, action potentials were only evoked/ recorded in the cells co-expressing JEDI-2P-kv and ChroME-ST (black)**. (e)** Raster plots from 3 further experiments in which 5 cells were targeted simultaneously. Black lines indicate the time at which a cell fired; the red lines indicate the imaging/photostimulation laser. All data were acquired using laser C fixed at 940 nm and camera A (See Supplementary Figure 1 and Supplementary Tables 1 and 2).

Next, we extended the unique capability of our approach of scanless two-photon voltage imaging to perform simultaneous two-photon photostimulation and imaging of multiple cells (Figure 8). In each experimental session, we first sequentially targeted cells that were expressing one or both constructs to quantify the probability of false positives (Figure 8a–c). We detected an increase in fluorescence when targeting cells only expressing ChroME-ST, due to excitation and detection of the nuclear-targeted fluorophore. No action potentials were identified in cells that were not co-expressing the two constructs but were detected optically in approximately fifty percent of co-expressing cells. We then simultaneously stimulated and imaged the same group of cells (Figure 8d) with no deterioration of SNR or −%ΔF/F_0_ of evoked and imaged action potentials (Figure 8d) and were able to determine the number and timing of action potentials evoked, and identify failures, during the stimulation period in multiple cells simultaneously (Figure 8e).

These results demonstrate that simultaneous, scanless two-photon voltage imaging and photostimulation can be performed in multiple cells simultaneously using high-energy, low-repetition rate lasers, using powers well-below the damage threshold.

## Discussion

In this work, we introduced scanless two-photon voltage imaging and performed high-contrast, high-resolution voltage imaging of single and multiple neurons expressing the newly developed GEVI JEDI-2P-kv. Due to the axial confinement conferred by temporal focusing we were able to perform high-contrast two-photon voltage imaging in densely labelled intact brain slices.

We performed a thorough characterisation of three, temporally focused, parallel excitation modalities (2P-TF-Gaussian, 2P-TF-GPC and 2P-TF-CGH) for scanless two-photon voltage imaging. A strong advantage of 2P-TF-Gaussian illumination (similarly to 3D-SHOT) is that it is the easiest and most cost-effective approach to implement. 2P-TF-Gaussian beams have been used for volumetric Calcium imaging^56,57^. However, it is the least photon-efficient approach. Conversely, the photon-dense speckle grains in 2P-TF-CGH spots result in efficient two-photon excitation and hence the highest SNR. In the case of limited power budget, 2P-TF-CGH is thus the optimal modality for scanless multitarget voltage imaging. However, since we observed higher photorecovery with 2P-TF-GPC and 2P-TF-Gaussian, these modalities are preferred for prolonged (continuous illumination for hundreds of milliseconds or more) recordings such as imaging spontaneous activity. Looking ahead, one of the primary advantages of 2P-TF-GPC is the capability of sculpting well-defined lateral shapes^36^ in order to target the most responsive regions of the cell membrane. In contrast with existing two-photon voltage imaging approaches, the high lateral resolution (0.1625 μm pixel size) of the experimental system presented in this manuscript would also be capable of sculpted, scanless two-photon voltage imaging of thin subcellular processes.

We used 2P-TF-GPC to demonstrate many of the theorized advantages of imaging neural activity with GEVIs by imaging action potentials (single-trial), subthreshold depolarizations and resolving single action potentials in high-frequency spike trains up to 125 Hz. We performed simultaneous imaging and electrophysiology to comprehensively characterize the performance of, and optimize, scanless two-photon voltage imaging with the genetically encoded indicator JEDI-2P-kv. Consistent with previous reports^30^, we found that it was generally possible to reduce the imaging speed down to 500 Hz (and consequently the required power) without a critical loss in the ability to determine the number and timing of action potentials. However, these imaging speeds reduce the accuracy of action potential detection for spike trains with frequencies > 100 Hz. The optimal imaging conditions will also depend on the characteristics of the specific GEVI used. In principle, a major advantage of voltage versus calcium imaging in neuroscience is the ability to detect sub-threshold changes in somatic membrane potential. In our current configuration, we found that imaging small sub-threshold signals required averaging data from up to 25 individual trials to reach a SNR above 1 for depolarizations larger than 0.5 mV. Whilst these findings highlight the challenges of detecting subthreshold unitary PSPs (< 2.5 mV) *in vivo* with current state-of-the-art GEVIs under two-photon excitation, they also indicate that such experiments ought to be possible using GEVIs optimized for sub-threshold voltage detection.

Since the current implementation of scanless two-photon voltage imaging requires continuous illumination, we carefully investigated whether the illumination conditions required to observe different aspects of neural activity induced any observable physiological perturbations. We found that single cells could be imaged using 100 fs, 80 MHz sources, at lower average powers than those commonly used for existing kilohertz scanning microscopes applied to two-photon voltage imaging^28,58,59^ and did not observe any changes in AP properties at these powers. However, we found that the latency of electrically induced APs was increased slightly at all powers tested, and irreversible thermal damage was revealed with 2P-TF-CGH at the highest powers tested using immunohistochemistry. In contrast, Caspase-3 staining did not reveal any non-linear damage at the investigated powers. In fact, we found that one of the biggest impediments to long-term voltage imaging was sample drift, a problem we imagine will be significantly more severe for scanless two-photon imaging during future *in-vivo* experiments. To overcome this problem, we plan to use a tandem construct containing the voltage indicator and a nuclear targeted, spectrally shifted, fluorescent reporter in order to track the sample drift and to dynamically update the co-ordinates of the multiplexed spots. Use of such a construct would also allow segmentation of the field of view and automated estimation of neuron location, which also ought to increase the accuracy of spot position relative to the somal membrane.

We also demonstrated that scanless two-photon voltage imaging could be performed with a much lower effective repetition rate (250 kHz) than used with existing kilohertz scanning microscopes. Even with this low-repetition rate laser, fluorescence was excited with more than 50x the number of pulses per voxel than for the scanning approaches. As a result, scanless two-photon voltage imaging is much more robust to fluctuations in output laser power than the scanning approaches. We demonstrated AP detection in single trials using 15–30 times lower average power than required using the high-repetition rate laser. Under these conditions, we did not find any histological evidence of thermal stress or physiological perturbations with whole-cell patch clamp recordings. As demonstrated in the case of two-photon optogenetics^55^, the major advantage of low-repetition rate lasers is that multiple targets can be illuminated simultaneously, whilst the average power delivered to the sample is kept below the thermal damage threshold, and much lower than the average powers used for existing kilohertz scanning microscopes. Specifically, in this work we were able to perform simultaneous two-photon voltage imaging of spontaneous activity in multiple neurons (up to 8) using a 100 fs, 250 kHz, 940 nm laser source (with between 200 – 600 mW exit power, corresponding to a maximum average power of 60 mW at the sample). In order to increase the number of target cells, it would be necessary to replace the laser source used in our experiments with a higher-power, low-repetition rate industrial light source such as existing Ytterbium-doped fibre lasers (1030 nm, > 10 W output power). In this work, we demonstrated that electrically evoked action potentials and spontaneous activity in hippocampal organotypic slices could be recorded by exciting JEDI-2P-kv at 1030 nm. Based on the results presented in this manuscript, with a 1030 nm Ytterbium-doped fibre source it would be feasible to record the voltage of 40 cells simultaneously (5 mW per cell) using the existing configuration for scanless two-photon voltage imaging, whilst remaining below the damage threshold (200 mW total average power).

The use of an SLM for holographic light multiplexing of the temporally focused, sculpted light resulted in a nominal FOV of 250 × 250 μm^2^. However, the effective FOV used for individual high-speed voltage imaging recordings was reduced in one dimension due to the maximum number of rows of pixels that could be read out at a given acquisition rate. We demonstrated that it was possible to perform voltage imaging throughout the 250 × 250 μm^2^ area and to combine data in post-processing, although this was not optimized on our prototype system, and increasing the lateral field of view is one of the most urgent future avenues of development. The field of view of scanless two-photon voltage imaging could be trivially increased by reducing the magnification of the detection axis, although this would not be suitable for all experiments. Furthermore, the sCMOS could be replaced with a detector capable of higher (full frame) readout speeds. A primary motivation behind the development of holographic light multiplexing was to enable multitarget photostimulation at axially distinct planes^43,60,61^. Although our system is also capable of targeting neurons in three-dimensions, we can currently only perform high-contrast imaging of a single plane. Performing scanless activity recordings from multiple planes simultaneously would require increasing the depth of field of the detection axis. For a very small number of discrete planes, this would be feasible using existing approaches, such as remote focusing^62,63^. More generally, camera-based volumetric voltage imaging will require the implementation of computational imaging approaches, such as variants of light-field microscopy, which have already been applied to imaging in scattering tissue^64^.

All experiments in this study were performed at relatively superficial depths (<50 μm) in scattering tissue where the expression pattern of JEDI-2P-kv was confined due to the approach used for viral delivery (bulk transduction). The next step will be to monitor the membrane potential of multiple neurons simultaneously *in-vivo*. It has already been demonstrated that temporal focusing preserves the profile and axial confinement of sculpted light up to 500 μm in scattering tissue for the three excitation modalities used in this work^38,45^, and camera detection has also been used to perform functional imaging with multi-spot excitation at depths up to 300 μm *in-vivo*^65,66^. Hence it ought to be possible to perform scanless two-photon voltage imaging in the upper cortical layer. Reaching deeper brain structures could be achieved by combining camera detection with excitation through graded index lenses (GRIN) lenses^67,68^ or via emerging computation approaches capable of overcoming scattering-induced ambiguity and of de-mixing the fluorescent transients emanating from different sources^69^.

We combined two-photon voltage imaging and optogenetics for the first time in proof-of-principle all-optical neurophysiology experiments. We capitalized on the overlapping excitation spectra of JEDI-2P-kv and the channelrhodopsin Chrome-ST to simultaneously evoke and record APs using a single beam. This approach could be incorporated into all-optical experiments to dynamically tune the incident power necessary for photostimulation and obtain the desired actuation on each of the targeted neurons *in situ*. In contrast to approaches relying on inferring neural activity from GCaMP fluorescence^70^, we demonstrated that voltage imaging provides a direct readout of the precise number and timing of optically-evoked action potentials with single spike precision at high spiking rates. The single beam approach to optically induce and read out neuronal activity will also be a major addition in connectivity mapping to non-invasively confirm the successful optical induction of an action potential in the potential pre-synaptic cell population. We demonstrated that scanless two-photon imaging can be performed with any of the existing modalities used for parallel two-photon photostimulation. As seen in our all-optical recordings (Figure 7d), the excitability of the targeted cells can vary between cells and over time, advising a confirmation of the pre-synaptic spike in each instance. In contrast to current connectivity mapping approaches, which do not confirm pre-synaptic spiking^71^ or use GECIs^70^, an approach using a voltage indicator would additionally reveal the precise timing of the pre-synaptic spike, which would facilitate correlation of the post-synaptic response and discrimination from noise. We anticipate that improved stoichiometric co-expression of the GEVI and the channelrhodopsin, possibly using a fusion-construct for tandem expression where they are covalently coupled, would facilitate the adoption of this approach.

The development of spectrally orthogonal voltage indicators and excitatory channelrhodopsins would facilitate the next-generation all-optical neurophysiology experiments. For example, it would be possible to record sensory-evoked activity patterns using voltage imaging, replay this activity using optogenetic stimulation and, also tune the excitation parameters in order to explore the logic and syntax of neural computation. Furthermore, this configuration would enable all-optical connectivity experiments whereby putative presynaptic neurons are stimulated optogenetically and sub-threshold post-synaptic responses are recorded optically. However, performing crosstalk-free all-optical neurophysiology experiments based on two-photon excitation is not trivial since the majority of two-photon compatible voltage indicators are optimally excited between 920 and 980 nm, a region of the electromagnetic spectrum where all commonly used channelrhodopsin variants are persistently activated^72^. While all-optical experiments with calcium indicators have been reported, similar results with GEVIs are more challenging due to the higher average powers required for high-SNR millisecond-timescale voltage imaging. The development of performant red-shifted genetically encoded voltage indicators, which could be combined with spectrally orthogonal blue-shifted channelrhodopsins, will remove these remaining challenges and fill an important gap in the optogenetic toolbox.

We anticipate that the description and thorough characterisation of scanless two-photon voltage imaging presented in this manuscript will motivate its application to deciphering the logic and syntax of neural circuits.

## Methods

### Experimental setup for performing two-photon voltage imaging with temporally focused, sculpted light

All two-photon voltage imaging presented in this manuscript was performed using the experimental setup presented in Supplementary Figure 1. In the schematic diagram, all reflective spatial light modulators (SLMs) are shown as transmissive for illustrative purposes. Path 1 was used to generate temporally focused, multiplexed, GPC (12 μm FWHM, 2PE) or low NA, apertured, Gaussian beams (12 μm FWHM, 2PE) respectively (upper path, Supplementary Figure 1). Path 2 (lower path, Supplementary Figure 1) was used to generate temporally focused holographic disks (12 μm FWHM, 2PE). Three different laser sources were used to acquire all data presented, referred to as Lasers A, B and C respectively throughout the manuscript. The specific source used to acquire each dataset is specified in each case and all experimental configurations used to acquire the data presented in each figure are summarised in Supplementary Tables 1 and 2. Laser A refers to a tuneable femtosecond source (Coherent Discovery, 80 MHz, 100 fs) tuned to 920, 940 or 1030 nm (as specified). Laser B refers to a femtosecond source with a fixed wavelength output (Spark Alcor, 4 W, 80 MHz, 100 fs, 920 nm). Laser C refers to a custom OPA pumped by an amplified laser, also with fixed wavelength output (Amplitude Satsuma Niji, 0.2 – 0.6 W, 250 kHz, 100 fs, 940 nm). By virtue of the removable mirrors indicated in Supplementary Figure 1, light from each of the lasers could be directed through path 1 or path 2 during a given experiment as indicated by the dashed lines in Supplementary Figure 1. In all cases, the average laser power at the sample plane was controlled using a half-wave plate (Thorlabs, WPHSM05-980) mounted on a motorised rotation mount (Thorlabs, PRM1Z8) in combination with a polarising beam splitter (PBS), (Thorlabs, CCM1-PBS253/M). Prior to each experiment, the efficiency of each path was measured. The power at the sample plane was recorded using a handheld power meter (Thorlabs, S121C) and the power of the s-polarised light exiting the PBS at each laser output was measured with a second power meter (Ophir, 30(150) A-BB-18, Nova II). The power of the s-polarised beam was monitored continuously during experiments and used to update the rotation of the half-wave plate to deliver the desired power at the sample plane during a given acquisition (calculated using the experimentally measured efficiency of each path). All half-wave plates were externally triggered prior to each acquisition. The output of lasers A and B was modulated by a mechanical shutter (Thorlabs, SH05R/M) or using a high-speed modulator (Thorlabs, OM6NH/M) whereas the output of laser C was gated directly. In all cases the external trigger was a TTL signal generated by pCLAMP (Molecular Devices, Sunnyvale, CA) controlling an acquisition system (Molecular Devices, Axon Digidata 1550B).

In path 1, a telescope formed of two lenses (L1, f = 80 mm, (Thorlabs, AC508-80-B) and L2, f = 300 mm, (Thorlabs, AC508-300-B), for GPC and L1, f = 80 mm, (Thorlabs, AC508-80-B) and L2, f = 200 mm, (Thorlabs, AC508-200-B) for low-NA Gaussian illumination) was used to expand and project the beam onto a spatial light modulator (SLM1), (Hamamatsu, LCOS 10468-07, 600 × 800 pixels, 20 μm pitch*)*. In the case of GPC, SLM1 was used to apply a π phase shift to the portion of the beam overlapping with the circular spot and a phase shift of zero elsewhere. The modulated beam was Fourier transformed by L3, f = 400 mm, (Thorlabs, AC508-400-B), resulting in a spatial displacement between the low and high spatial frequency components of the field in the Fourier plane. The low spatial frequency components were selectively phase shifted by π using a phase contrast filter (PCF) with 60 μm radius (Double Helix Optics, custom design) positioned in the Fourier plane of L3. The spatial frequencies were recombined in the image plane by L4, f = 300 mm, (Thorlabs, AC508-300-B). For more details, refer to^36^. For parallel two-photon excitation using a low NA Gaussian beam, the corrective phase mask provided by the manufacturer was displayed on SLM1 and the PCF was displaced from the optical path as indicated in Figure 1. A blazed diffraction grating (Richardson Gratings, 600 lines/mm) located at the focal plane of L4, a conjugate image plane, disperses the different spectral frequency components of the ultrafast beam as required for temporal focusing. The grating was oriented at the blaze angle to maximize light throughput. The dispersed beam was collimated in one direction and Fourier transformed in the orthogonal direction by L5, f = 500 mm, (Thorlabs, AC508-500-B), resulting in an asymmetric “line” illumination of SLM2 (Hamamatsu, LCOS 10468-07, 600 × 800 pixels, 20 μm pitch), as described previously^61^. For this work, SLM2 was used for 2-dimensional multiplexing of the beam, although 3-dimensional multiplexing would be possible. Phase masks were generated using a weighted Gerchberg-Saxton algorithm as described previously^48,73^. For all data presented in Figures 1, 2 and 3, SLM2 was used to displace the sculpted light from the optical axis and the zeroth-diffraction order, which was removed using a physical beam block positioned in a conjugate image plane. Lenses 6 (f = 500 mm, (Thorlabs, AC508-500-B)) and 7 (f = 300 mm, (Thorlabs, AC508-300-B)) were used to de-magnify the beam to the back focal plane of the objective lens (Nikon, CFI APO NIR, x40, 0.8 NA, f = 5 mm) which projected the light (and re-combined the different spectral frequency components) onto the focal plane. A half-wave plate was included downstream of SLM2 in path 1 to convert s-polarised light to p-polarised.

In path 2, a Galilean beam expander formed of two lenses (L8, f = −75 mm, *(*Thorlabs, LC1258-B) and L9, f = 500 mm, (Thorlabs, AC508-500-B)), expanded the beam onto SLM3, (Hamamatsu, LCOS X13138-07, 1272 × 1024 pixels, 12.5 μm pitch). For the single cell experiments, 5 holograms designed to generate a single 12 μm holographic spot (located in the same position) were computed prior to each session. For each recording, one of these phase masks was randomly selected and displayed on the SLM in order to minimize any effects of the variable speckle distribution on the resulting dataset. The holograms displayed on SLM3 were designed to generate multiple 12 μm holographic spots targeted to chosen neurons throughout the field of excitation following the calibration procedure outlined in the Supplementary Information. All holograms were calculated using an iterative Gerchberg-Saxton algorithm^74^. The zeroth diffraction order was removed using a physical beam block positioned in a conjugate image plane. The modulated beam was Fourier transformed by L10 (f = 750 mm, (Thorlabs, AC508-750-B)), to form the holographic disks in a conjugate image plane where a blazed grating (Thorlabs, GR50-0610, 600 lines/mm) was located. A 2” diffraction grating was used to maximise the field of excitation. The diffraction grating was oriented perpendicular to the optical axis and the illumination angle was chosen such that the first diffraction order of the central wavelength propagated along the optical axis. This orientation did not coincide with the blaze angle, and hence was not the most efficient^23^ but was allowed the temporal focusing plane of each of the holographic disks to coincide with the focal plane of the objective lens. A pair of telescopes comprised of L11 (f = 500 mm, (Thorlabs, AC508-500-B)), L12 (f = 300 mm, (Thorlabs, AC508-300-B)), L7 (f = 300 mm, (Thorlabs, AC508-300-B)) and the objective lens (Nikon, CFI APO NIR, x40, 0.8 NA, f = 5 mm, water) was used to de-magnify and relay the holographic spots on to the sample plane. In experiments where non-temporally focused spots were used (1030 nm excitation), the diffraction grating was replaced by a mirror.

Excitation paths 1 and 2 were combined prior to the tube lens (L7) using a polarising beam splitter (Thorlabs, PBS253). The linear polarisation of the light exiting the objective was changed using a half-wave plate following the PBS. For each modality, the rotation of the half-wave plate was set to that which was found to maximise the two-photon excited JEDI-2P-kv fluorescence.

SLM1 was also used to optimise the alignment of the GPC path by translating the position of the π phase disk relative to the centre of the incident Gaussian beam and adding tip/tilt/defocus phases to translate the position of the focus with respect to the PCF filter. SLMs 1, 2 and 3 were also used to correct for system aberrations by adjusting the coefficients of Zernike modes (evaluated at the centre of each SLM pixel) in order to maximise the efficiency, uniformity and contrast of two-photon excited fluorescence excited in a thin rhodamine layer.

In all experiments, fluorescence was captured using a simple widefield detection axis comprised of a microscope objective (Nikon, CFI APO NIR, x40, 0.8 NA, f = 5 mm, water), a tube lens (TL), (Thorlabs, TTL200-A) and a scientific complementary metal-oxide semiconductor (sCMOS) camera (Hamamatsu ORCAFlash 4.0 or Photometrics Kinetix, as summarised in Supplementary Table 1). For both sCMOS detectors, the pixel size at the sample plane was 0.1625 μm and 1×1 binning was used for all experiments. The fluorescence was separated from the excitation light using a dichroic mirror (Semrock #FF705-Di01, 70 × 50 mm). Widefield, single photon, epi-fluorescence excitation was accomplished by means of two LED sources (Thorlabs M490L4, 490 nm to excite JEDI-2P-kv fluorescence and Thorlabs M430L5, 430 nm to excite BFP fluorescence), filtered by bandpass excitation filters (Semrock FF02-482/18, FF01-414/46) and focused onto the back focal plane of the objective lens by means of an achromatic lens (Thorlabs f = 50 mm). Long exposure times (1s) and low excitation power densities were used to acquire all images based on single photon fluorescence. For single photon or dual-colour imaging (for instance during all-optical experiments), fluorescence was filtered using either a quad-band filter (Chroma ZET405/488/561/640) or individual bandpass filters (Chroma ET525/50, ET605/7). Infrared light used for two-photon excitation of fluorescence was blocked from the camera using a shortpass filter (Semrock #FF01-750sp).

Data were acquired using a control scheme based on custom scripts written to control *micro-manager 2.0 Gamma*^75^ from Python via Pycro-manager^76^. All experiments were controlled using two desktop computers running Windows 10. During voltage imaging experiments, the micro-manager acquisition engine was bypassed. Data from the camera was streamed directly to disk on one of the acquisition computers. The first step of any experiment was to acquire 2 (JEDI-2P-kv and transmitted light) or 3 (JEDI-2P-kv, ChroME-ST (H2B-BFP2) and transmitted light) widefield images of the sample. These images were used to select targeted cells during a given experiment. The centroids of all targeted cells were written to file for all experiments. All widefield images presented in this text are background subtracted for visualization purposes (rolling ball background subtraction, ImageJ, rolling ball radius 50 pixels). All the voltage imaging data presented in this manuscript were acquired in “dynamic range”, 16-bit mode, which meant that a maximum of 266 rows of pixels could be acquired at 1 kHz (43 × 250 μm^2^ FOV). In practice we used an exposure time of 1 ms resulting in an effective acquisition rate of 980 Hz following 0.02 ms readout (similarly for the recordings referred to as 500 and 750 Hz in the manuscript). For single cell experiments (corresponding to data presented in Figures 2–5), data were only acquired for a square region with diameter less than 266 pixels centred on a given cell. For the multi-cell experiments, neurons were grouped to find the maximal number which could be imaged within 266 pixels. The relative centroids of all targeted cells within this cropped region, and the upper left-hand coordinate of each cropped region were written to file for all experiments. These coordinates were used to “stitch” data from sequential acquisitions into a single dataset. In “dynamic range” mode, the field of view is inversely proportional to the exposure time, such that we could acquire data from 532 rows (86 × 250 μm^2^ FOV) at 500 Hz. The field of view could be increased by a factor of 6 using “speed” mode, where data is read out at 8-bit. The camera was triggered using a 5 V, TTL signal generated by pCLAMP (Molecular Devices, Sunnyvale, CA) controlling an acquisition system (Molecular Devices, Axon Digidata 1550B). During experiments, widefield images were visualised using the open-source image viewer Napari and voltage imaging traces were visualised using pyqtgraph.

All fluorescence traces were analysed using the same analysis pipeline written in Python, as outlined in the main text and Supplementary Information, derived from^50^. When multiple cells were imaged simultaneously, the (known) centroid of the excitation spot in camera co-ordinates was used to crop a rectangular region of interest (ROI) surrounding each cell (generally 100 × 100 pixels). In rare cases where the ROIs of independent cells overlapped, a region of each independent cell was identified manually. Individual cells were then defined according by regression of each pixel in the ROI against the average fluorescence trace of the manually segmented pixels.

### Preparation of CHO cells

CHO cells were acquired from Sigma (Sigma, 85050302) and cultured in T25 flasks (Falcon, 353107) in a medium consisting of DMEM-F12 + Glutamax (Fisher, Gibco^™^ 10565018), supplemented with 10% SBF (Fisher, Gibco^™^ 10500-064) and 1% penicillin/streptomycin (5000 U ml^−1^). Cells were passaged every 2–3 days. Prior to each experiment, cells were seeded on coverslips (Fisher, 10252961) in 24-well plates (50 000 cells/ml). After 24 hours, cells were transiently transfected with a plasmid using the Jet prime kit (Ozyme, POL101000015) (Table 1). The medium was then replaced after 4 hours. Experiments were performed 48 hours post transfection.

### Electrophysiology for scanless two-photon voltage imaging in CHO cells

48 hours post transfection, whole-cell voltage clamp recordings of JEDI-2P-kv-expressing CHO cells were performed at room temperature (21 – 23°C). An upright microscope (Scientifica, SliceScope) was equipped with a far-red LED (Thorlabs, M660L4), oblique condenser, microscope objective (Nikon, CFI APO NIR, 40X, 0.8 NA), tube lens (Thorlabs, TTL200-A), and an sCMOS camera (Photometrics, Kinetix, or Hamamatsu, Flash4.0) to collect light transmitted through the sample. Patch clamp recordings were performed using an amplifier (Molecular Devices, Multiclamp 700B), a digitizer (Molecular Devices, Digidata 1550B) at a sampling rate of 10 kHz and controlled using pCLAMP11 (Molecular Devices). Cells were continuously perfused with artificial cerebrospinal fluid (ACSF) comprised of 125 mM NaCl, 2.5 mM KCl, 1.5 mM CaCl_2_, 1 mM MgCl_2_, 26 mM NaHCO_3_, 0.3 mM ascorbic acid, 25 mM D-glucose, 1.25 mM NaH_2_PO_4_. Continuous aeration of the recording solution with 95% O_2_ and 5% CO_2_, resulted in a final pH of 7.4 (measured). Borosilicate pipettes (with filament, OD: 1.5 mm, ID: 0.86 mm, 10 cm length, fire polished, WPI) were pulled using a Sutter Instruments P1000 puller, to a tip resistance of 3.5–6 MΩ. Pipettes were filled with an intracellular solution consisting of 135 mM K-gluconate, 4 mM KCl, 4 mM Mg-ATP, 0.3 mM Na_2_-GTP, 10 mM Na_2_-phosphocreatine, and 10 mM HEPES (pH 7.35). All membrane potentials reported in this manuscript are Liquid Junction Potential (LJP) corrected by –15 mV (measured). Recordings were compensated for capacitance (Cm) and series resistance (Rs) to 70 % (Cm = 11.6 ± 4.7 pF; Rs = 16.1 ± 8.2 MΩ; mean ± s.d.). Only recordings with an access resistance below 35 MΩ were included in subsequent analysis.

All experiments were performed with laser A, except data presented in Supplementary Figure 20 where laser C was used.

For protocols 1 and 2 (Figure 2), JEDI-2P-kv-expressing CHO cells were patched and clamped at −55 mV and 3, 100 mV steps were applied under either continuous (3 s, power density: 0.88 mW μm^−2^, corresponding to 100 mW per cell) or strobed illumination (200 ms every 2.5 s, power densities ranging from 0.66 to 1.55 mW μm^−2^ (75 to 175 mW per cell), as specified in the main text). The fluorescent responses to the depolarization steps were simultaneously recorded at 100 Hz. For protocol 3, JEDI-2P-kv-expressing CHO cells were patched and clamped at −75 mV to mimic the resting potential of neurons in the dentate gyrus of hippocampal organotypic slices. A 20 Hz train of 10, 3 ms, 100 mV steps was electrically induced, and the fluorescent response to different scanless illumination methods was recorded simultaneously (500 ms, power density: 1.33 mW μm^−2^, corresponding to 150 mW per cell, 1 kHz acquisition).

The ability to record voltage responses using JEDI-2P-kv under 1030 nm illumination was assessed using protocol 2 with a holographic spot (12 μm diameter, not temporally focused, power density: 0.4 mW μm^−2^, corresponding to 45 mW per cell, Supplementary Figure 19).

For data presented in Supplementary Figure 20, ChroME-ST expressing cells were patched in whole cell voltage clamp configuration at −55 mV and photocurrents in response to 17.5 ms pulses of light (power densities ranging from 0.02 to 0.04 mW μm^−2^, corresponding to 2.5 to 5 mW per cell) were recorded.

### Preparation of hippocampal organotypic slice cultures for validating scanless two-photon voltage imaging of neuronal activity using JEDI-2P-kv

All experimental procedures were conducted in accordance with guidelines from the European Union and institutional guidelines on the care and use of laboratory animals (Council Directive 2010/63/EU of the European Union). Hippocampal organotypic slices were prepared from mice (Janvier Labs, C57Bl6J) at postnatal day 8 (P8). Hippocampi were sliced with a tissue Chopper (McIlwain type 10180, Ted Pella) into 300 μm thick sections in a cold dissecting medium consisting of GBSS (Sigma, G9779) supplemented with 25 mM D-glucose, 10 mM HEPES, 1 mM Na-Pyruvate, 0.5 mM α-tocopherol, 20 nM ascorbic acid, and 0.4% penicillin/streptomycin (5000 U ml^−1^).

After 30 – 45 min of incubation at 4 °C in the dissecting medium, slices were placed onto a porous membrane (Millipore, Millicell CM PICM03050) and cultured at 37 °C, 5% CO2 in a medium consisting of 50% Opti-MEM (Fisher 15392402), 25% heat-inactivated horse serum (Fisher 10368902), 24% HBSS, and 1% penicillin/streptomycin (5000 U ml^−1^). This medium was supplemented with 25 mM D-glucose, 1 mM Na-Pyruvate, 20 nM ascorbic acid, and 0.5 mM α-tocopherol. After three days in-vitro (DIV), the medium was replaced with one containing 82% neurobasal-A (Fisher 11570426), 15% heat-inactivated horse serum (Fisher 11570426), 2% B27 supplement (Fisher, 11530536), 1% penicillin/streptomycin (5000 U ml^−1^), which was supplemented with 0.8 mM L-glutamine, 0.8 mM Na-Pyruvate, 10 nM ascorbic acid and 0.5 mM α-tocopherol. This medium was removed and replaced once every 2–3 days.

Slices were transduced with AAV9_hSyn_JEDI-2P_Kv2.1 at DIV 3 by bulk application of 1 μl of virus per slice (see Table 2). Experiments were performed between DIV 7 and 15.

### Electrophysiology for validating scanless two-photon voltage imaging of neuronal activity using JEDI-2P-kv in hippocampal organotypic slices

At DIV 10–15, whole-cell patch clamp recordings of JEDI-2P-kv expressing granule cells in DG were performed at temperatures varying between 31–35°C. During experiments, slices were perfused with ACSF as previously described. This extracellular solution was supplemented with 1 μM AP5 (Abcam, ab120003) and 1 μM NBQX (Abcam, ab120046) in all experiments except for the spontaneous activity recordings (Figures 5–6, Supplementary Figures 14 and 18). Patch pipettes were filled with intracellular solution (see above).

Neurons were held at −75 mV in voltage clamp configuration and recordings were compensated for capacitance (Cm) and series resistance (Rs) to 70 % (Cm = 21 ± 6.3 pF; Rs = 19.2 ± 8.5 MΩ; mean ± s.d.). In current clamp configuration, neurons were injected with some current (less than 100 pA) if necessary to maintain their resting membrane potential to −75 mV. In the latter configuration, bridge potential was corrected (Bridge potential = 13.9 ± 4.2 MΩ; mean ± s.d.).

Neurons were first patched in whole-cell voltage clamp configuration. Protocol 1 was then performed to confirm that the fluorescence of the patched cell was voltage responsive (Supplementary Figure 12).

The ability to record single action potentials in neurons (Figure 3c and d) was assessed by electrically triggering a 1 Hz train of 50 action potentials with short latency and jitter under strobed illumination (10 ms, power densities ranging from 0.66 to 1.55 mW μm^−2^, corresponding to 75 to 175 mW per cell) while recording at three different acquisition rates (500 Hz, 750 Hz and 1 kHz). Action potentials were triggered by injecting 700–900 pA currents for 2 ms.

Then, to assess the ability to record fast spike trains in neurons, trains of 10 action potentials from 25 to 125 Hz were electrically induced under illumination at different power densities (from 0.66 to 1.55 mW μm^−2^, corresponding to 75 to 175 mW per cell) and recorded at acquisition rates varying between 500 Hz, 750 Hz and 1 kHz. The amount of current injected was one that was sufficient to evoke 10 action potentials at each of the different spike trains. Recordings where one action potential was missing in the electrophysiological trace were dismissed. 125 Hz was found to be the limit at which the granule cells could spike in our conditions (Figure 3e–f and Supplementary Figure 13).

For data presented in Figure 4, 6, 20 ms steps separated by 30 ms and ranging from 0 to 2.5 mV (in 0.5 mV steps) were induced in JEDI-2P-kv expressing neurons under strobed illumination (40 ms centered around the steps, power density: 1.33 mW μm^−2^, corresponding to 150 mW per cell) while recording the fluorescence response at 1 kHz. This was repeated 50 – 75 times.

To record spontaneous activity (Figure 5), JEDI-2P-kv expressing neurons were patched and their membrane potential was monitored under continuous illumination for 30 s at a power density of 1.33 mW μm^−2^ (150 mW per cell) while recording the fluorescent response at 1 kHz. Cells were not patched for the recordings presented in Supplementary Figure 14. The same protocol was repeated to perform long-term voltage imaging of JEDI-2P-kv expressing neurons for a maximum of 20 min with a dark period of <10 s in between each recording (due to limitations in data transfer rates). To overcome this issue, we performed longer recordings (1 min) at a lower acquisition rate (500 Hz) with the same power density (1.33 mW μm-2, corresponding to 150 mW per cell). This allowed us to shorten the dark period in between recordings to < 5 s (Supplementary Figure 15).

The axial resolution of JEDI-2P-kv (Supplementary Figure 12) was measured by electrically triggering an action potential and measuring the fluorescence response while displacing the objective in the z axis (from +50 to −50 μm, in 5 μm steps). The lateral resolution was measured (from +20 to −20 μm, in 2 μm steps) by mechanically moving the sample in the x-y axis.

To measure the performances of JEDI-2P-kv under 1030 nm illumination, hippocampal organotypic slices were infected with a mixture of AAV1_EF1a_DIO_JEDI-2P_Kv2.1_WPRE and AAV9_hSyn_Cre_WPRE_hGH (see Table 2) at DIV 3 in order to get a sparser expression. Isolated expressing cells in the dentate gyrus were then patched in whole cell current clamp configuration and illuminated with a holographic spot (12 μm diameter, not temporally focused) at 1030 nm (power density: 1.21 mW μm^−2^, corresponding to 137 mW per cell). Single action potentials and spontaneous activity recordings were obtained as described previously (Supplementary figure 19).

### Preparation of hippocampal organotypic slices for two-photon actuation and imaging of neural activity using ChroME-ST and JEDI-2P-kv

All animal procedures followed national and European animal care guidelines (Directive 2010/63/EU) and institutional guidelines on animals used for research purposes. Hippocampal organotypic slice preparations were prepared as described in reference^77^ with a few modifications. Briefly, hippocampi were extracted from P5-P8 C56Bl/6J mouse pups sacrificed by decapitation. The dissection was carried out in filter sterilized (0.2 μm pore size) ice cold medium containing: 248 mM sucrose, 26 mM NaHCO_3_, 10 mM glucose, 4 mM KCl, 5 mM MgCl_2_, 1 mM CaCl_2_, 2 mM kynurenic acid and 0.001 % phenol red saturated with 95 % O_2_ / 5 % CO_2_. Transverse slices of 300 – 400 μm thickness were cut with McIlwain Tissue Chopper using double edge stainless steel razor blades. Using a plastic transfer pipette, undamaged slices were individually transferred onto the small pieces of PTFE membrane (Millipore FHLP04700) placed on membrane inserts (Millicell PICM0RG50) in the 6-well-plate containing 1 mL pre-warmed culture medium. The slices were cultured at 37 °C and 5 % CO_2_ in antibiotic free culture medium consisting of 80 % MEM and 20 % Heat-inactivated horse serum supplemented with 1 mM L-glutamine, 0.01 mg/ml Insulin, 14.5 mM NaCl, 2 mM MgSO_4_, 1.44 mM CaCl_2_, 0.00125 % Ascorbic acid and 13mM D-glucose. The culture medium was partially replaced with fresh, 37 °C warmed culture medium every 3 days.

Various titrations were tested to achieve sufficient levels of expression of both sensor and actuator. When slices were transduced with both viruses on the same day, we observed a reduction in the expression of JEDI-2P-kv. Furthermore, overexpression-mediated apoptosis was observed in some cases when slices were transduced with both viruses simultaneously. The best results were obtained by transducing slices with JEDI-2P-kv first, followed a week later by ChroME-ST which resulted in strong co-expression of both proteins (Figure 8a). However, in general we found that the expression levels of both proteins were more variable when the two constructs were co-expressed than when either construct was expressed independently.

Slices were transduced firstly with AAV9-hSyn-JEDI-2P-Kv2.1 at DIV 3 and secondly with AAV9_Camk2a_ChroME-ST_P2A_H2B_tagBFP2 (provided by H. Adesnik, University of California, Berkeley, USA) at DIV 10 by bulk application of 1 μl of virus per slice (Table 2). Channelrhodopsin-expressing cells were visualized using stable expression of an H2B-BFP2 fusion, which resulted in nuclear localized BFP2 fluorescence. Experiments were performed between DIV 13 and 17.

To characterize the performances of ChroME-ST (Figure 7), ChroME-ST and JEDI-2P-kv co-expressing granule cells in DG were patched in whole-cell current clamp configuration. 5, 17.5 ms pulses of light at 5 Hz were applied at different power densities ranging from 0 to 0.09 mW μm^−2^ (0 to 10 mW per cell) to photo-evoke action potentials. The fluorescent responses were recorded at an acquisition rate of 1 kHz. The latency and jitter of light-evoked action potentials, respectively defined as the mean and standard deviation of the time between the onset of stimulation and the peak of the action potential, were measured using the same protocol. The axial resolution of ChroME-ST was measured using a similar protocol, while displacing the spot axially by mechanically moving the objective from +75 to −50 μm, in 5 μm steps in the vicinity of the cell (from +55 μm to −10 μm) and then in 10 μm steps (Figure 7 and Supplementary Figure 20). For each cell, the power density was increased until a spike was detected optically in at least one of five repeats. The final set of power densities used was between 0.02 – 0.08 mW μm^−2^ (2.5 – 9 mW) per cell.

### Immunostaining

Immunostaining was performed on hippocampal organotypic slices to assess the potential non-linear photodamage induced by two different laser sources (A and C) during our experiments.

In the case of laser A, slices expressing JEDI-2P-kv were illuminated with a holographic spot (12 μm diameter, temporally focused, power densities between 0.66 – 1.55 mW μm^−2^, corresponding to 75 – 175 mW per cell) in the dentate gyrus. The illumination protocol consisted of 50, 10 ms pulses of light, using the same protocol used to record single action potentials (see previous section), repeated on > 15 cells per region illuminated. A negative control (no illumination) and a positive control where a whole region was continuously illuminated for 30 min (power density: 1.64 mW μm^−2^, corresponding to 185 mW per cell) were also performed.

Slices expressing JEDI-2P-kv and ChroME-ST were illuminated with 5 holographic spots generated with laser source C (12 μm diameter, power densities ranging between 0.02 – 0.09 mW μm^−2^ (2.5 – 10 mW per cell), 45 μm separation), and moved laterally across 120 μm in 20 μm steps. The illumination protocol used to characterize the ChroME-ST (see previous section) was repeated 5 times at each position, and the hologram was recomputed each time.

After experiments, slices were immediately fixed in PFA 4% for 3–5 min. Permeabilization of the tissue was performed by incubation of the slice in a solution comprising of Triton X-100 in PBS (0.5 %) for 12 hours at 4 °C. Non-specific sites were then blocked by incubation in a blocking solution (BSA 20 % in PBS) for 4 hours at room temperature (21–23 °C).

Slices were incubated with primary antibodies diluted in a solution of BSA 5 % in PBS (Table 3) overnight at 4 °C and placed in a solution of BSA 5 % in PBS on a horizontal shaker for 10 minutes to wash off excess antibodies. This process was repeated three times.

Slice were then incubated with species-appropriate secondary antibodies conjugated to Alexa fluor 555 (to detect anti-activated-Caspase-3 immunostaining) and Alexa fluor 647 (to detect anti-HSP70/72 immunostaining) diluted in the same solution as the primary ones, for 3–4 hours at room temperature (21–23 °C). They were then washed again following the same process, but in PBS only.

Slices were immediately mounted in Fluoromount-g mounting medium (Southern Biotech, 0100–01) to be imaged using confocal microscopy (Olympus FV3000, 20X magnification, 0.8 NA, pixel size 0.6214 μm, λ 488, 561, 640 nm). The same imaging parameters were used for all experimental conditions.

### Statistics

All experiments were repeated for at least two (and generally many more) independent passages of cells, transfections or infections. The Shapiro test (scipy.stats.shapiro) was used to test whether data were normally distributed. For normally distributed data, the paired or unpaired two-tailed students t-test was used to compare two independent samples. The non-parametric Mann-Whitney *U*-test (scipy.stats.mannwhitneyu) was used to compare two samples in the case when either or both samples were found not to be normally distributed. ‘n’ refers to the number of independent biological replicates, as stated in each figure caption and summarized in Supplementary Table 2. A statistical comparison was deemed significant if the p-value was less than 0.05. For all figures * denotes p<0.05, ** denotes p<0.01 and *** denotes p<0.0001. All results reported in the manuscript are communicated as the mean value ± standard deviation of at least three technical replicates unless otherwise stated. As specified, error bars in plots denote either the standard deviation or the standard error. All biological replicates were included in each estimate. Estimation stats were performed using the Python package dabestr^78^.

## Figures and Tables

**Table 1:** List of plasmids

Plasmid	Transfection ratio
pAAV_hSyn_JEDI-2P_GSS3_Kv2.1	0.75 μg DNA: 1.5 μl transfectant
pAAV_CamKIIa_ChRoME-ST_P2A_H2B_BFP	0.75 μg DNA: 1.5 μl transfectant

**Table 2:** List and final titres of viruses

Virus	Final titer (vg ml^−1^)
AAV9_hSyn_JEDI-2P_GSS3_Kv2.1	3.12 × 10^13^
AAV9_CamKIIa_ChRoME-ST_P2A_H2B_BFP	4.46 × 10^12^
AAV1_EF1a_DIO_JEDI-2P_Kv2.1_WPRE	2.36 × 10^13^
AAV9_hSyn_Cre_WPRE_hGH	2.3 × 10^11^

**Table 3:** List and dilutions of antibodies used for immunostaining

Antibody	Supplier	Reference	Species	Working dilution
HSP70 / HSP72	Enzo Life Science	ADI-SPA-810-D	Mouse	1:400
Cleaved Caspase-3 (Asp175)	Cell Signaling	9661	Rabbit	1:250
Anti-mouse, Alexa fluor 647	Thermofisher Scientific	A21235	Goat	1:500
Anti-rabbit, Alexa fluor 555	Thermofisher Scientific	A21429	Goat	1:500
